# Synthesis of bimannich base with thiazole and its corrosion inhibition effect on H_2_S and CO_2_ at high temperature

**DOI:** 10.1186/s13065-021-00784-9

**Published:** 2021-11-03

**Authors:** Li Zhuoke, Cao Jun, Mao Ting, Ni Dan

**Affiliations:** 1 Southwest Oil and Gas Field Branch, Research Institute of Natural Gas Technology, Petrochina Ltd, Chengdu, Sichuan China; 2 National Energy High Sour Gas Reservoir Exploitation R&D Center, Chengdu, Sichuan China; 3 High Sulfur Gas Exploitation Pilot Test Center of CNPC, Chengdu, Sichuan China; 4 Northwest Sichuan Division, PetroChina Southwest Oil Gas Field Company, Jiangyou, 621714 Sichuan China

**Keywords:** Thiazole, Bimannich base, Corrosion inhibitor, High temperature resistance

## Abstract

A bimannich-based TZBM containing a thiazole ring was obtained by synthesis of mannich bases. TZBM featured a stable structure at 260 °C, and corrosion inhibition effect on carbon steel in a gas–liquid environment with Cl^−^ + H_2_S + CO_2_ at 180 °C. By analyzing the weight loss of steel exposed to different TZBM concentrations, the coverages of the inhibitor adsorbed on the surfaces were determined, and the results conformed to Langmuir isotherm model. Furthermore, the negative Gibbs free energy indicated that the adsorption was a spontaneous process.

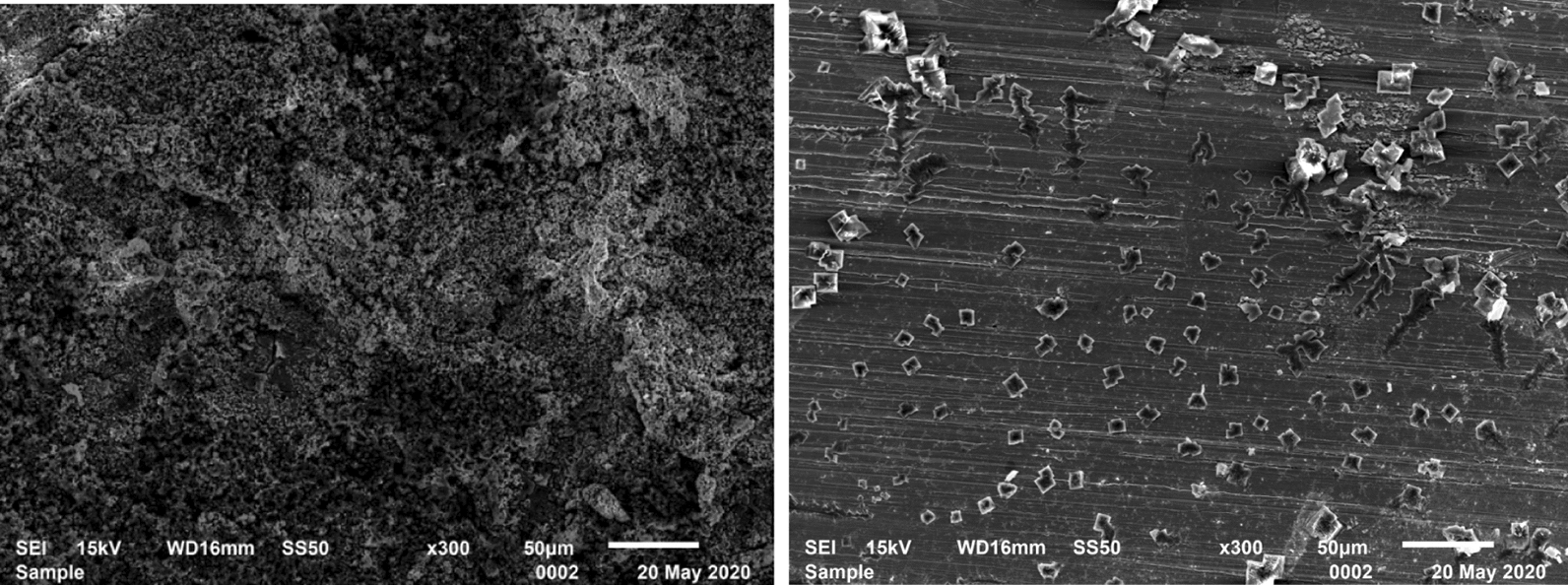

## Introduction

The sour gas and sour water are highly corrosive to the metal materials when passing through the pipelines. For example, as to the pipelines of sour gas wells, corrosion inhibitors are applied on the metal pipes to improve the gas transmission environment [1–3], ensure safe production and extend the service life of metal pipes. The research field of corrosion inhibitors used in the oil–gas gathering and transmission system is mature because of the mild conditions [4–7]. However, as for the corrosion inhibitors used in downhole, due to the more complex conditions and higher temperatures in the deeper places, the effect of the existing inhibitors is reduced.

The most popular corrosion inhibitor for H_2_S and CO_2_ is imidazoline corrosion inhibitor, which is widely used in oil and gas fields, especially in pipelines, and is proven to be effective. The main method to get imidazoline corrosion inhibitors is the two-step dehydration of polyvinyl polyamines and long-chain acids. The first step of dehydration is carried out at 140–160 °C, and the second step at 200–230 °C [8–14]. Reaction conditions are very important. In addition, organic compounds containing hetero-atoms such as N, O and S are also proven corrosion inhibitors for metals and alloys [15–20]. The Mannich base is usually used as the corrosion inhibitor for well acidizing treatments, requiring only one step with a reaction temperature of 60–80 °C and mild conditions [21–26]; however, it is seldom used for H_2_S and CO_2_ environment. Studies have shown that the synthetic bis-Mannich base outperforms the Mannich base.

In this paper, a bis-Mannich base with thiazole synthesized in the laboratory turned out to be an effective corrosion inhibitor for the steel exposed to corrosive liquid containing Cl^−^ + H_2_S + CO_2_, so this compound is an option for the corrosion protection for the H_2_S and CO_2_ environment.

## Experiment

### Substances

2-Acetylthiazole(AR), Benzaldehyde(AR), Tetraethylenepentamine(AR), Hydrochloric Acid (AR), Absolute Ethanol (AR), Petroleum Ether (AR), NaCl (AR), Carbon dioxide (99.999%), Nitrogen (99.999%), Hydrogen sulfide (99.99%).

### Synthesis

**Scheme 1:**
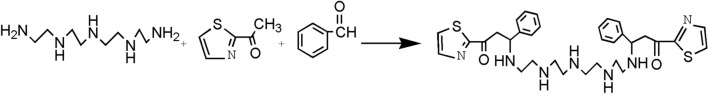
Synthetic route of TZBM

One-pot synthesis was implemented as follows to get bimannich-based TZBM. First, benzaldehyde was added to the ethanol (solvent) in the three-neck flask. Tetraethylenepentamine was then slowly added, followed by 2-acetylthiazole. The molar ratio of the three solutes was 2:2:1. Concentrated hydrochloric acid was slowly added till the pH value is 4 ~ 5. The solution was heated till the temperature reaches 80 °C for condensation and reflux. 6 h later, a reddish-brown liquid was obtained, and crude TZBM could be obtained after distillation under reduced pressure. Finally, it was recrystallized with absolute ethanol 2–3 times to obtain a purer product. Reaction equation is illustrated in Scheme 1.

### Thermogravimetric analysis

The thermogravimetric analysis (TGA) of TZBM was carried out in the nitrogen atmosphere using Mettler Dsc823 supplied by METTLER TOLEDO. The starting temperature was 40 °C, and the heating rate was 10 °C/min.

### Gravimetric analysis

The size of the specimens for weight loss measurements was 30 mm × 15 mm × 3 mm. Surface finishing was conducted with emery paper (400−1000 Grit).

Atmospheric pressure experiment: ①Nitrogen was added for 8 h, to remove the oxygen in NaCl solution (5.0L, 5.0%). The deoxygenated NaCl solution was halved in two containers. ②CO_2_ was gently added to the deoxygenated NaCl solution (2.5L, 5.0%) for 8 h to get the CO_2_-saturated solution. At the same time, H_2_S was gently added to the deoxygenated NaCl solution (2.5L, 5.0%) for 8 h to get the H_2_S-saturated solution. ③The CO_2_-saturated solution and the H_2_S-saturated solution were mixed at 1:1 to get the corrosion solution. ④Two specimens were immersed into the corrosion solutions with/without TZBM at different concentrations in oil bath at 80 °C, and oxygen was removed by nitrogen. ⑤72 h later, the specimens were taken out, rinsed with distilled water, washed with the membrane-removing acid solution, alcohol, and petroleum ether, dried, and weighed using an analytical balance having a readability down to 0.01 mg.

High-temperature and high-pressure test: 650 mL 5.0% NaCl solution and a calculated amount of corrosion inhibitor were added into the autoclaves, and two specimens were hung into the autoclaves and immersed in the liquid. Nitrogen was blown in for 6 h, to remove oxygen. The autoclaves were sealed. After 2.0 MPa CO_2_ and 1.0 MPa H_2_S were added in the autoclaves, nitrogen was added till the total pressure is 10 MPa. Two specimens were immersed into the corrosion solutions with/without TZBM at different concentrations at 180 °C. 72 h later, the specimens were taken out, rinsed with distilled water, washed with the membrane-removing acid solution, alcohol, and petroleum ether, dried, and weighed using an analytical balance having a readability down to 0.01 mg.

### Observation of corrosion morphology

The surfaces of the specimens undergoing the high-temperature and high-pressure test without being rinsed were observed with a scanning electron microscope (JSM-6510), which could help analyze the corrosion inhibition effect of TZBM on the specimens in gas–liquid environment exposed to Cl^−^ + H_2_S + CO_2_. And EDS test by the scanning electron microscope was used for quantitative analysis of element content, to determine the formation of corrosion products.

## Results and discussion

### Structure characterization

**Fig.1:**
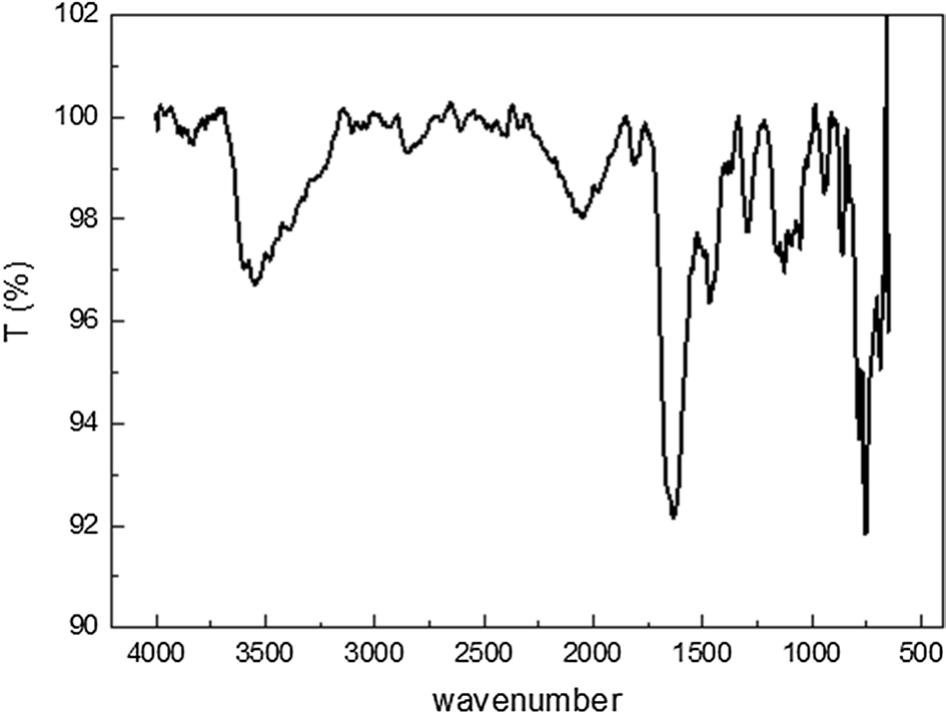
Infrared characterization spectrum of TZBM

Figure 1 shows the infrared spectroscopy of TZBM. The graph presents a single peak at 3542 cm^−1^, which is the characteristic peak of the stretching vibration of the secondary amine N–H bond. The stretching vibration peak of the methylene C-H bond appears at 2862 cm^−1^, and the carbonyl group becomes connected to the heterocyclic ring at 1646 cm^−1^. The C=N double bond in the thiazole has the stretching vibration peak at 1461 cm^−1^, and fingerprint regions (745 cm^−1^ and 675 cm^−1^ ranges) are characteristic peaks of CH deformation vibrations monosubstituted by benzene ring. Meanwhile, there is no obvious narrow double peak at 3500 ~ 3400 cm^−1^, indicating that there is no functional group of primary amines; there is no strong peak at 1720 cm^−1^, indicating that there is no C=O double bond of aldehyde. It shows that the reaction is completed and the target product is successfully synthesized.

**Fig.2:**
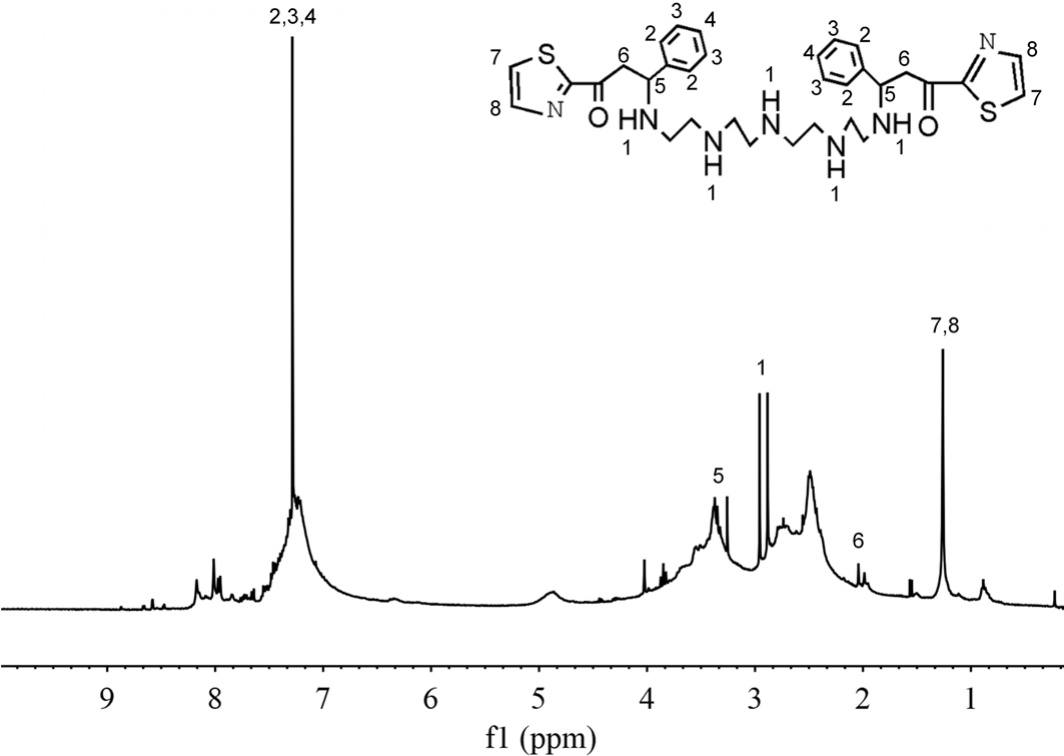
The 1 H NMR (400 MHz, D2O) spectrum of TZBM

The ^1^ H NMR in Fig. 2 further verifies the synthesis of TZBM. The 2.88 ppm point shows the chemical shifts of protons of polyamines marked as 1. The peak of the chemical shift of protons of –CH– of benzene backbones is found at 7.27 ppm (marked as 2, 3 and 4). The peak at 3.28 ppm (marked as 5) is the peak of the chemical shift of protons of the –CH– bonded with N atom of the polyamines. 2.06 ppm shows the chemical shifts of the protons of the –CH_2_– bonded with C=O marked as 6. The peak of the chemical shift of protons of Thiazole rings is found at 2.25 ppm (marked as 7 and 8). The molecular structure of synthesized AHAPAM obtained from ^1^ H NMR spectrum is consistent with that obtained from the FTIR spectrum.

### Thermogravimetric analysis

**Fig.3:**
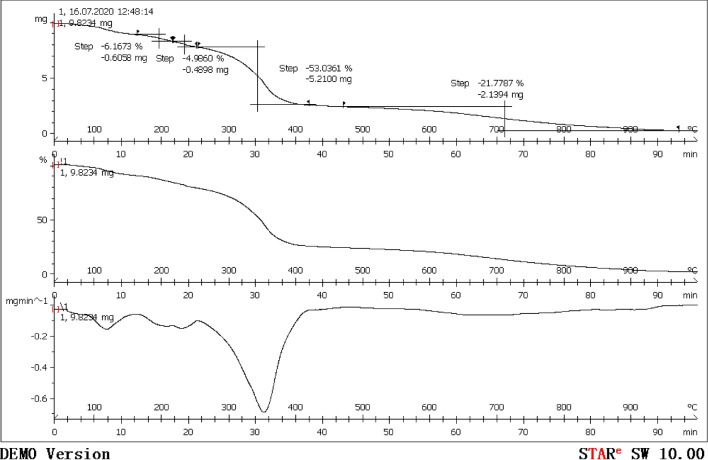
Thermogravimetric analysis of TZBM

Figure 3 shows that the initial weight of the tested TZBM sample is 9.8234 mg. As the temperature gradually increases, the weight of TZBM firstly is unchanged and then drops sharply and finally is unchanged again. In the temperature range of 260–400 °C, the weight of TZBM drops sharply by 53%. The rate of weight loss first increases and then decreases, and reaches a maximum value of about 0.8 mg/min at about 360 °C. These indicate that the chemical structure of TZBM is stable at 260 °C, and that the structure becomes unstable and the chemical bond breaks when the temperature exceeds 260 °C. Therefore, TZBM is of excellent temperature resistance when used in the natural gas gathering pipelines and downhole.

### Weight loss measurements

Table 1 shows the inhibitor efficiency and corrosion rates obtained from weight loss measurements with different concentrations of the inhibitor in the prepared corrosion solution at 80 ± 0.1 °C. With the weight loss calculated, the inhibitor efficiency η is calculated using the following equation:

1 $$ \eta = {{{\text{(R}}0 - {\text{R)}}} \mathord{\left/ {\vphantom {{{\text{(R}}0 - {\text{R)}}} {{\text{R}}0}}} \right. \kern-\nulldelimiterspace} {{\text{R}}0}} \times 100{\text{\% }} $$

**Table 1:** Inhibition efficiency for various concentrations of TZBM for the corrosion of steel in the prepared corrosion solution obtained from weight loss measurements

C_TZBM_ (ppm)	Corrosion rates (mm/a)	Inhibitor effificiency (%)
Blank	2.1897	−
50	0.0780	96.44
100	0.0747	96.58
200	0.0742	96.61
300	0.0656	97.0
400	0.0479	97.81

where R and R_0_ are rates of corrosion (mm/a) with/without inhibitor.

**Fig.4:**
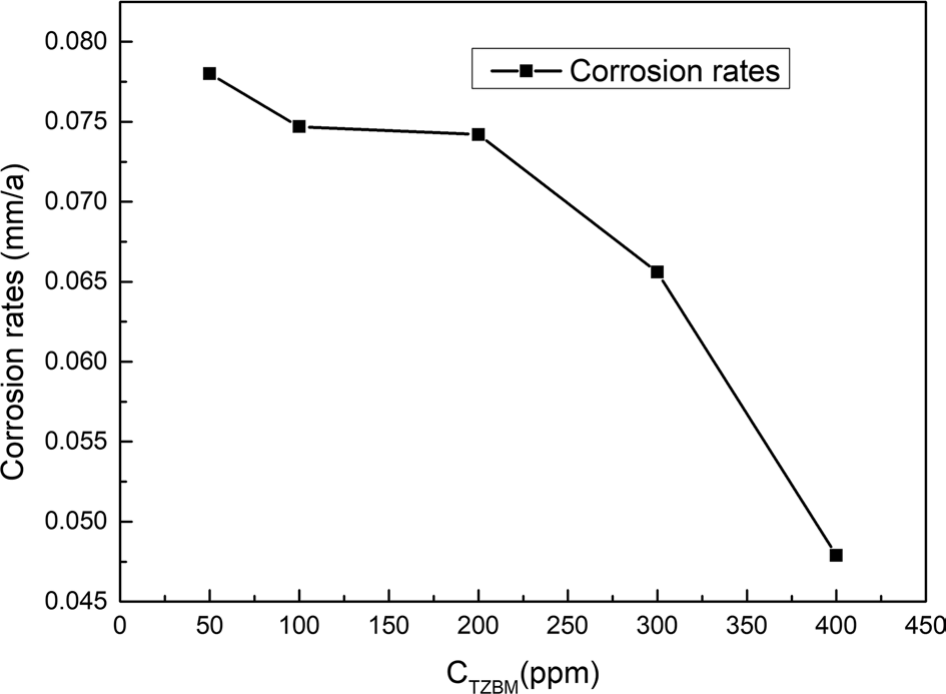
Influence of corrosion inhibitor concentration on corrosion rate

Figure 4 shows that corrosion rate decreases with increase in TZBM concentration. In the concentration range of 50-–200 ppm, the corrosion rate reduces slowly. It means that if the amount of corrosion inhibitor is small, the specimen surface cannot be fully covered. When the concentration of the corrosion inhibitor exceeds 300 ppm, the corrosion rate is significantly reduced. At 400 ppm, the corrosion rate is only 0.0479 mm/a, indicating that when the corrosion inhibitor fully covers the surface of the specimen, it can effectively block the corrosive medium and protect the steel.

### Adsorption isotherm

To understand the mechanism of corrosion inhibition, it is necessary to know the adsorption behavior of the organic adsorbate on the metal surface.

The main assumptions of the Langmuir adsorption model proposed by Irving Langmuir are the following: ①The adsorption and desorption on the surface are reversible and reach a dynamic equilibrium; ②The surface of the adsorbent still has residual forces for attraction. The adsorption capacity is the same; ③The adsorption occurs at the single-molecule site, and the maximum surface coverage *θ* is 1; ④The adsorbed medium have no influence on each other. Therefore, the Langmuir isotherm equation can be expressed as Eq. 2 [27–29].

2 $$ \theta = {{{\text{K}}\alpha } \mathord{\left/ {\vphantom {{{\text{K}}\alpha } {(1 + }}} \right. \kern-\nulldelimiterspace} {(1 + }}{\text{K}}\alpha ) $$

where, *θ* is the surface coverage, K is the thermodynamic equilibrium constant of adsorption, and *ɑ* is the activity of corrosion inhibitor.

For the assumed simple-molecule adsorptive behavior of TZBM, inhibition efficiency *η* can replace surface coverage *θ*. And in low concentration. The activity of corrosion inhibitor *ɑ* can be replaced by the concentration *C*. Therefore, Eq. 3 is derived from Eq. 2.

3 $$ C/\eta = C + 1/K $$

Considering the influence of other factors, we adapt Eq. 3 by multiplying a correction factor f, and Eq. 4 is as follows.

4 $$ C/\eta = {\text{f}}C + {\text{f}}/K $$

**Fig.5:**
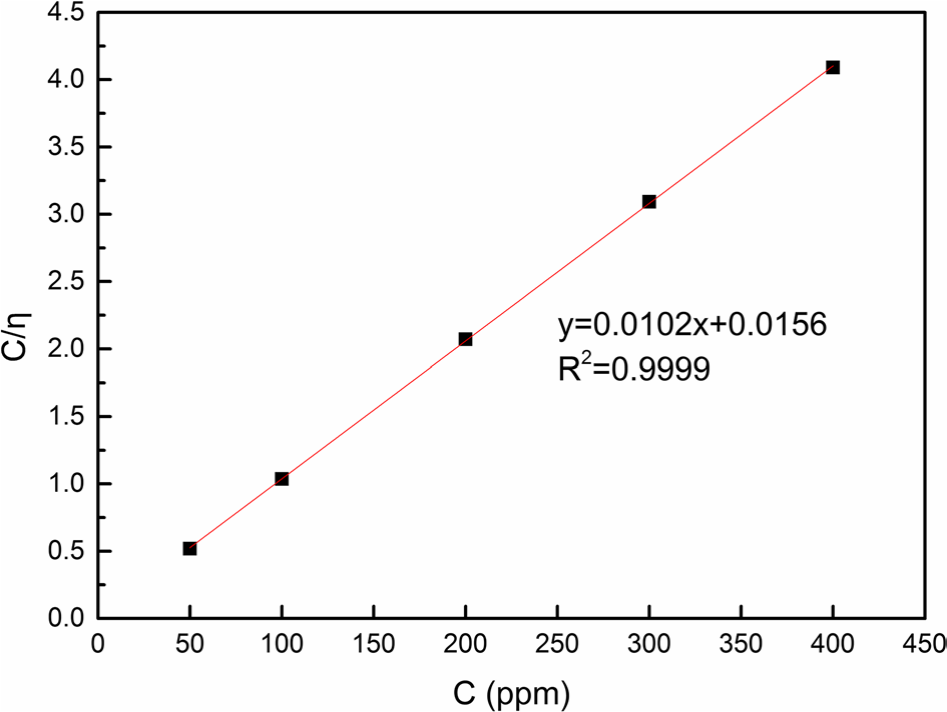
Langmuir’s isotherm for adsorption of TZBM on the steel surface in the prepared corrosion solution

According to Table 1, the surface coverage is tested to allow fitting of a suitable adsorption isotherm. The plot of *C*/*η* versus *C* (Fig. 5) yields a straight line with nearly unit slope, proving that the adsorption of the TZBM from the prepared corrosion solution on the steel obeys the Langmuir adsorption isotherm [30, 31].

The equilibrium adsorption constant K obtained from the Langmuir plot is about 0.65 × 10^3^ l/mol. From Van’t Hoff equation (Eq. 5), ΔG is − 19.02 kJ/mol, which indicates that TZBM is strongly adsorbed on the steel surface, and that this adsorption process is spontaneous.

5 $$ \Delta {\text{G}} = - {\text{RTlnK}} $$

**Fig.6:**
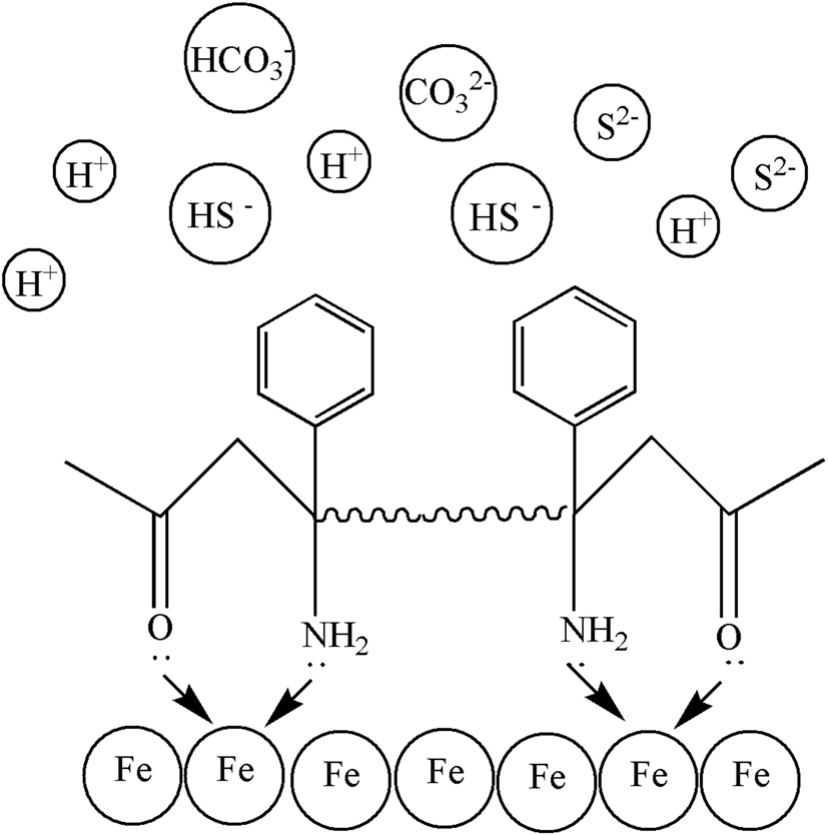
Schematic diagram of adsorption mechanism

From this analysis, TZBM prevents corrosive media from passing through single molecules adsorbed on the surface of the steel. The principle of adsorption is that the N and O atoms in the molecule contain a lone pair of electrons, which can enter the hybrid orbital of the iron atom in the steel to form a coordination bond. Because of the special structure of TZBM, this coordination bond just establishes a stable six-membered ring structure, and each molecule can form two adsorption points, as shown in Fig. 6, so that TZBM is more firmly adsorbed on the surface of steel, which improves its high temperature resistance.

### High-temperature and high-pressure test

**Fig.7:**
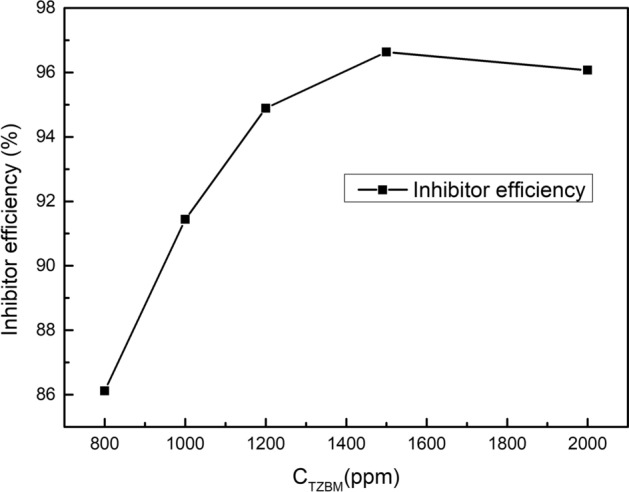
Influence of corrosion inhibitor concentration on inhibitor efficiency in the high-temperature and high-pressure test

Since temperature has a great influence on the corrosion rate, to ensure the corrosion inhibition effect of TZBM, the concentration of TZBM was increased to 1500 ppm in the high-temperature and high-pressure test. Table 2 shows the results of high-temperature and high-pressure test for corrosion inhibition performance of TZBM at different concentration. The trends of corrosion at different concentrations is shown in Fig. 7. As the concentration increases, the corrosion inhibition efficiency gradually increases until the concentration is 1500 ppm. Because the molecular movement is violent at high temperature, it is more difficult for the corrosion inhibitor to be adsorbed on the surface of the steel. Adding more corrosion inhibitor to the solution will help increase the amount of corrosion inhibitor adsorbed on the surface of the steel to increase the corrosion inhibition efficiency. And when the corrosion inhibitor exceeds a certain concentration, the steel surface cannot absorb more corrosion inhibitor, therefor the corrosion inhibition efficiency will not increase.

**Table 2:** The results of high temperature and high pressure test for corrosion inhibition performance of TZBM

C_TZBM_ (ppm)	Corrosion rates (mm/a)	Inhibitor efficiency (%)
Blank	1.6156	−
800	0.2242	86.12
1000	0.1382	91.44
1200	0.0826	94.89
1500	0.0544	96.63
2000	0.0635	96.07

**Fig.8:**
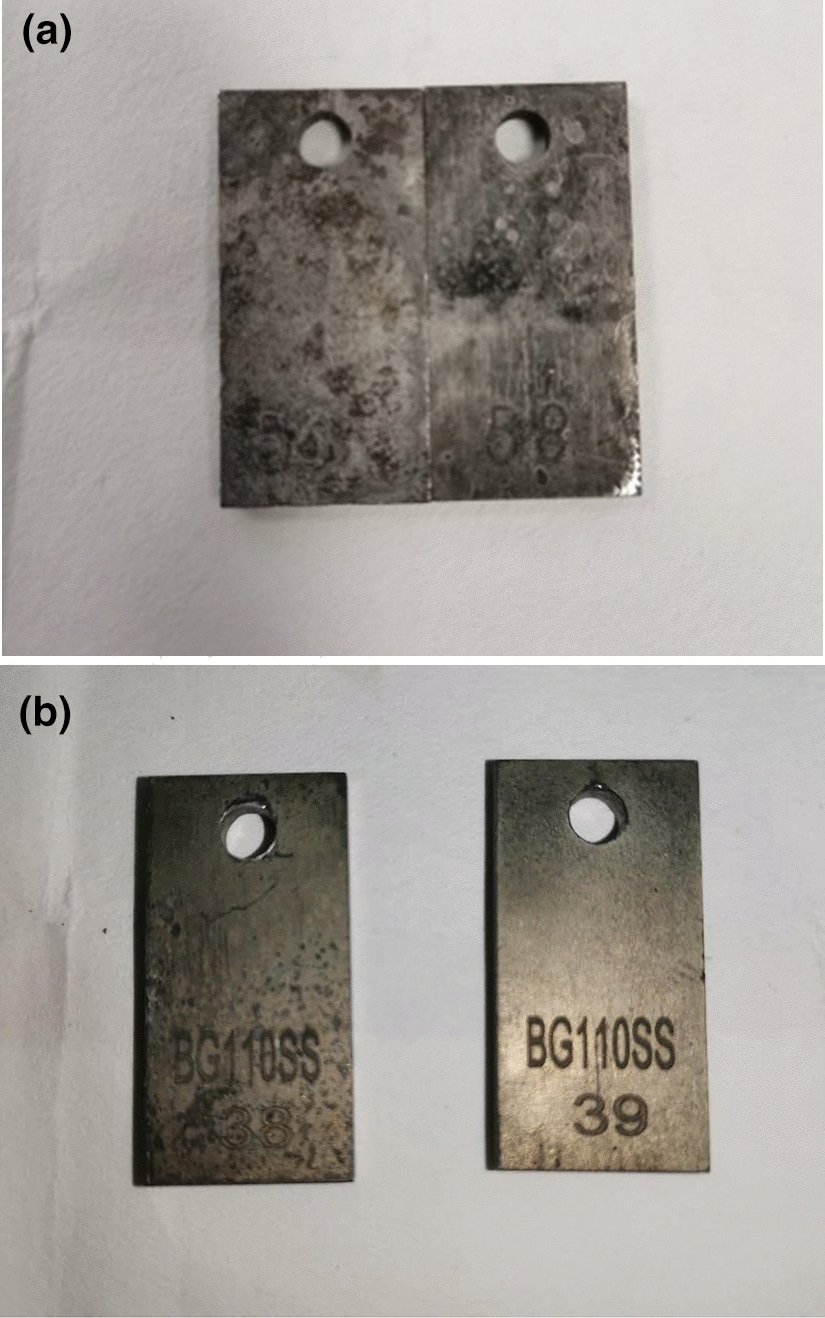
**a **The photo of the appearance of after-cleaning specimens without inhibitor; **b** The photo of the appearance of after-cleaning specimens with inhibitor TZBM

Figure 8 is the photo of the appearance of after-cleaning specimens with and without inhibitor, respectively. The high-temperature and high-pressure test proves that TZBM has good corrosion inhibition performance at high temperature.

### Corrosion morphology observation

**Fig.9:**
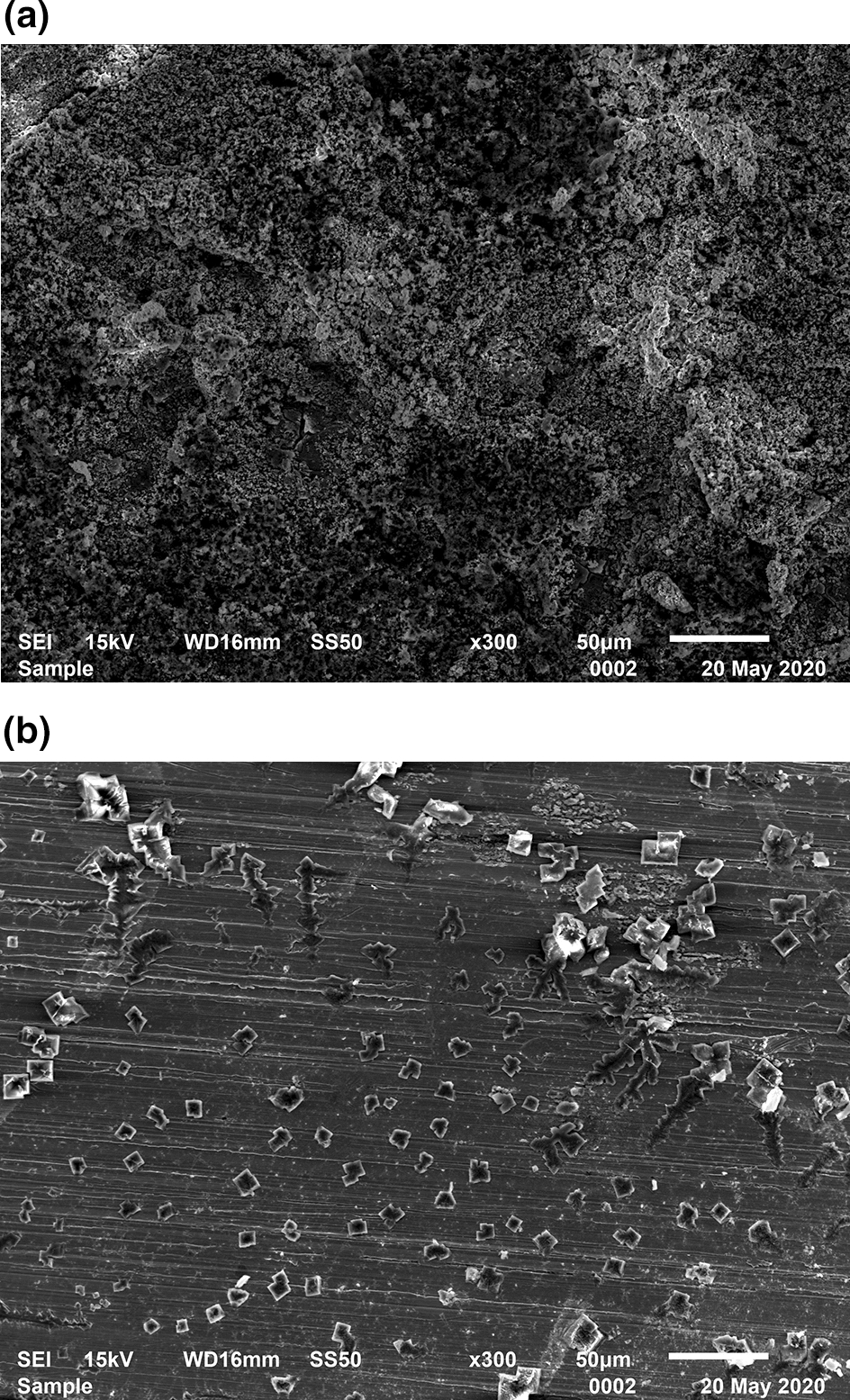
**a** Corrosion morphology of the surface of the specimen which from the test without inhibitor; **b** Corrosion morphology of the surface of the specimen which from the test with 1500 ppm inhibitor

Figure 9 shows the micro corrosion morphology of the specimens after the high-temperature and high-pressure test without being rinsed. There are large quantities of overlapping loose structures on the surface of the specimen from the test without inhibitor TZBM. The overlapping loose structures should be the corrosion products of steel. Differently, some crystalline objects can be observed on a flat surface of the specimen from the test with 1500 ppm inhibitor TZBM. Those crystalline objects should be NaCl precipitating from the corrosion solution.

Table 3 shows the distinguish of corrosion products on the surface in the corrosion solutions with/without TZBM. From the EDS test, the surface of the specimen from blank test has 28.95% atom S, but with TZBM there is only 4.43%. It means TZBM significantly reduced the production of iron-sulfur compounds.

**Table 3:** The percentage of atoms on the surface of the specimen from the test without/with 1500 ppm inhibitor

Element	C	O	Na	S	Cr	Fe
The percentage of atoms (blank)	14.09	18.63	2.50	28.95	0.22	35.61
The percentage of atoms (1500 ppm TZBM)	12.55	19.39	5.72	4.43	0.85	55.87

Through the observation of corrosion morphology, the result of adsorption behavior research is confirmed. TZBM can indeed adsorb stably on the surface of steel to block corrosive medium at high temperature.

## Conclusions

- The bis-Mannich-based TZBM containing thiazole structure is stable at the temperature below 260℃.
- TZBM inhibits the corrosion of steel in a gas–liquid environment containing Cl− + H_2_S + CO_2_.
- According to the weight loss experiment, the higher the concentration of inhibitor, the higher the inhibition efficiency.
- The adsorbed inhibitor molecules on the steel surface blocks corrosive medium and therefore inhibits corrosion. Adsorption of the inhibitor conforms to the Langmuir isotherm model.
- Enough TZBM forms a stable protective layer on the surface of the steel in the corrosive solution at 180 °C, and the corrosion rate of the steel is lower than 0.076 mm/a. This shows that TZBM is an excellent corrosion inhibitor for high-temperature environment.

## Data Availability

All data generated or analyzed during this study are included in this published article.

## Abbreviation

TZBM: The synthetic bimannich-based containing a thiazole ring in this study
